# Supplementary material for: MetaRibo-Seq measures translation in microbiomes
Source: Nat Commun. 2020 Jun 29;11:3268. doi: 10.1038/s41467-020-17081-z (PMC7324362; doi:10.1038/s41467-020-17081-z)
Supplement: Supplementary file 10 — Supplementary Data 7 [file 41467_2020_17081_MOESM10_ESM.zip › File2/Confidence_VeryHigh_Taxonomy/132373_out.krona.html]

Javascript must be enabled to view this page.

members
magnitude
magnitudeUnassigned
count
unassigned
taxon
rank

132373\_out

25

25
superkingdom
2

2
phylum
1239

186801
class
2

2
order
186802

186803
2
family

2
genus
2039240


SRS020869\_contig\_number\_contig-100\_5513.157358SRS064276\_contig\_number\_35753
160404
2
species

976
1
phylum

200643
1
class

171549
order
1

171552
family
1

838
1
genus

60133

SRS015941\_contig\_number\_15834
1
species

32066
phylum
22

203490
class
22

203491
22
order

22
family
203492

22
genus
848

860

SRS011140\_contig\_number\_contig-100\_2516.166773SRS012983\_contig\_number\_12802SRS017381\_contig\_number\_contig-100\_7782.74581SRS022625\_contig\_number\_1804SRS023557\_contig\_number\_contig-100\_193.75025.75025SRS024138\_contig\_number\_contig-100\_8640.95521SRS042589\_contig\_number\_18732SRS048411\_contig\_number\_19613SRS052518\_contig\_number\_contig-100\_3544.166668SRS053854\_contig\_number\_13685SRS054687\_contig\_number\_15602SRS056622\_contig\_number\_22803SRS064423\_contig\_number\_contig-100\_9751.72370SRS076868\_contig\_number\_contig-100\_5661.95211SRS097905\_contig\_number\_30341SRS104165\_contig\_number\_contig-100\_2526.207825SRS143088\_contig\_number\_contig-100\_1777.91176SRS144512\_contig\_number\_20078SRS147541\_contig\_number\_contig-100\_27849.68261SRS147712\_contig\_number\_3478SRS148150\_contig\_number\_contig-100\_7721.102056SRS148973\_contig\_number\_18762
species
22
